# Supplementary material for: Vision Screening Assessment (VISA) tool: diagnostic accuracy validation of a novel screening tool in detecting visual impairment among stroke survivors
Source: BMJ Open. 2020 Jun 11;10(6):e033639. doi: 10.1136/bmjopen-2019-033639 (PMC7295409; doi:10.1136/bmjopen-2019-033639)
Supplement: Author's manuscript [file bmjopen-2019-033639.draft_revisions.pdf]

BMJ Open

BMJ Open is committed to open peer review. As part of this commitment we make the peer review history of every article we publish publicly available.

When an article is published we post the peer reviewers' comments and the authors' responses online. We also post the versions of the paper that were used during peer review. These are the versions that the peer review comments apply to.

The versions of the paper that follow are the versions that were submitted during the peer review process. They are not the versions of record or the final published versions. They should not be cited or distributed as the published version of this manuscript.

BMJ Open is an open access journal and the full, final, typeset and author-corrected version of record of the manuscript is available on our site with no access controls, subscription charges or pay-per-view fees (http://bmjopen.bmj.com).

If you have any questions on BMJ Open's open peer review process please email info.bmjopen@bmj.com

BMJ Open

The Vision Screening Assessment (VISA) tool - diagnostic accuracy validation of a novel screening tool in detecting visual impairment among stroke survivors.

Journal:	*BMJ Open*
Manuscript ID	bmjopen-2019-033639
Article Type:	Original research
Date Submitted by the Author:	14-Aug-2019
Complete List of Authors:	Rowe, Fiona; University of Liverpool, Health Services Research<br>Hepworth, Lauren; University of Liverpool, Health Services Research<br>Howard, Claire; University of Liverpool, Health Services Research<br>Bruce, Alison; Bradford Institute for Health Research, ; University of York, Health Sciences<br>Smerdon, Victoria; Arrowe Park Hospital, Orthoptics<br>Payne, Terry; University of Liverpool, Computer Science<br>Jimmieson, Phil; University of Liverpool, Computer Science
Keywords:	VISA, Screening, STROKE MEDICINE, Visual impairment, Orthoptics, Detection

SCHOLARONE™
Manuscripts

I, the Submitting Author has the right to grant and does grant on behalf of all authors of the Work (as defined in the below author licence), an exclusive licence and/or a non-exclusive licence for contributions from authors who are: i) UK Crown employees; ii) where BMJ has agreed a CC-BY licence shall apply, and/or iii) in accordance with the terms applicable for US Federal Government officers or employees acting as part of their official duties; on a worldwide, perpetual, irrevocable, royalty-free basis to BMJ Publishing Group Ltd ("BMJ") its licensees and where the relevant Journal is co-owned by BMJ to the co-owners of the Journal, to publish the Work in this journal and any other BMJ products and to exploit all rights, as set out in our licence.

The Submitting Author accepts and understands that any supply made under these terms is made by BMJ to the Submitting Author unless you are acting as an employee on behalf of your employer or a postgraduate student of an affiliated institution which is paying any applicable article publishing charge ("APC") for Open Access articles. Where the Submitting Author wishes to make the Work available on an Open Access basis (and intends to pay the relevant APC), the terms of reuse of such Open Access shall be governed by a Creative Commons licence – details of these licences and which Creative Commons licence will apply to this Work are set out in our licence referred to above.

Other than as permitted in any relevant BMJ Author's Self Archiving Policies, I confirm this Work has not been accepted for publication elsewhere, is not being considered for publication elsewhere and does not duplicate material already published. I confirm all authors consent to publication of this Work and authorise the granting of this licence.

For peer review only

The Vision Screening Assessment (VISA) tool - diagnostic accuracy validation of a novel screening tool in detecting visual impairment among stroke survivors.

Fiona J Rowe[1,2,3], Lauren R Hepworth[1,3,4], Claire Howard[1,3], Alison Bruce[4], Victoria Smerdon[5], Terry Payne[6], Phil Jimmieson[6]

1, Department of Health Services Research, University of Liverpool, UK

2, Department of Ophthalmology, Walton Centre for Neurology and Neurosurgery, Liverpool, UK

3, Department of Orthoptics, Salford Royal NHS Foundation Trust, Salford, UK

4, Bradford Institute for Health Research, Bradford Teaching Hospitals NHS Foundation Trust, Bradford, UK

5, Department of Orthoptics, Wirral University Teaching Hospital NHS Foundation Trust, Liverpool, UK

6, Department of Computer Science, University of Liverpool, UK

Address for correspondence:

Prof Fiona Rowe

Department of Health Services Research

Waterhouse Building Block B, 2nd floor,

University of Liverpool,

1-5 Brownlow Street,

Liverpool L69 3GL

E: rowef@liverpool.ac.uk

T: 0151 7944956

Disclosures: The authors report no conflict of interest for this study.

Funding: This research received no specific grant from any funding agency in the public, commercial or not-for-profit sectors.

Word count: 4187

Cover title: Visual impairment screening assessment tool (VISA)

Number of figures: 1

For peer review only - http://bmjopen.bmj.com/site/about/guidelines.xhtml

Number of tables: 4

Keywords: VISA; Screening; Stroke; Visual impairment; Orthoptics; Detection

Author contributions: FR provided oversight for the study and led the writing of the paper. FR, LH, CH, VS and AB contributed to data collection, reviewing the draft paper and approving the final version. TP and PJ contributed to the development of VISA app software and its testing, and reviewed the draft paper and approved the final version.

Data access; Data can be accessed via direct contact with the lead author.

For peer review only

Abstract

Purpose: Screening for visual problems in stroke survivors is not standardised. Visual problems that remain undetected or poorly identified can create unmet needs for stroke survivors. We report the validation of a new Vision Impairment Screening Assessment (VISA) tool intended for use by the stroke team to improve identification of visual impairment in stroke survivors.

Methods: We conducted a prospective case cohort comparative study in four centres to validate the VISA tool against a specialist reference vision assessment. VISA is available in print or as an app (MHRA regulatory approved); these were used equally for two groups. Both VISA and the comprehensive reference vision assessment measured case history, visual acuity, eye alignment, eye movements, visual field and visual inattention. The primary outcome measure was the presence or absence of visual impairment.

Results: Two hundred and twenty two stroke survivors were screened. Specialist reference vision assessment was by experienced orthoptists. Full completion of screening and reference vision assessment was achieved for 201 stroke survivors. VISA print was completed for 101 stroke survivors; VISA app was completed for 100. Sensitivity and specificity of VISA print was 97.67% and 66.67% respectively. Overall agreement was substantial; K=0.648. Sensitivity and specificity of VISA app was 88.31% and 86.96% respectively. Overall agreement was substantial; K=0.690. Lowest agreement was found for screening of eye movement and near visual acuity.

Conclusions: This validation study indicates acceptability of VISA for screening of potential visual impairment in stroke survivors. Sensitivity and specificity were high indicating the

For peer review only - http://bmjopen.bmj.com/site/about/guidelines.xhtml

accuracy of this screening tool. VISA is available in print or as an app allowing versatile uptake across multiple stroke settings.

Strengths and limitations of this study

- Validation of the VISA screening tool in this prospective study shows improved detection accuracy for detection of stroke-related visual impairment
- The study included clinicians involved in stroke care who are not specialists in vision problems and lack formal eye training.
- Where early visual impairment detection occurs, this facilitates prompt referral with fewer false positives and negatives.
- Through process evaluation, clinicians reported acceptability of the VISA screening tool for is use in screening for presence of vision problems in stroke survivors.
- The VISA screening tool may further be of potential use for visual screening in other care settings such as neuro-rehabilitation.

Introduction

The prevalence of overall visual impairment has been estimated at 65% with varying prevalence reported for specific types of visual impairment (1-3). Figures for incident new onset visual impairment following stroke are placed at about 60% (4). Given the estimated 100,000 new onset strokes per annum in the UK there are sizeable numbers of stroke survivors living with stroke-related visual impairment (5).

Visual impairment constitutes a considerable comorbidity of stroke. Visual impairment, on its own or in addition to other stroke-related disabilities, can cause significant impact to quality of life (6). For many, it results in inability or altered ability to undertake many aspects of daily activities with impact on return to work, participation in hobbies and family life, and can lead to social isolation, altered mood and depression (7-9). Interventions for stroke-related visual impairment are well established (10) but require referral to appropriate eye care services, which is facilitated through orthoptic service routes (11). Where visual impairment is identified, this facilitates optimisation of other therapy and early access to vision rehabilitation.

For peer review only - http://bmjopen.bmj.com/site/about/guidelines.xhtml

There are issues with how best to identify the presence of visual impairment through stroke team vision screening and specialist vision assessment (12). Even with screening measures in place there are also issues reported with provision of care and access to vision services for stroke survivors who have been identified as having vision problems (13).

The overall aim of this study was to validate the Vision Impairment Screening Assessment (VISA) tool which uses simple established assessments of visual function coupled with detailed instructions. Our objectives were to test VISA, available in print or as a software application, against a reference of a specialist vision assessment to determine sensitivity, specificity, predictive values and inter-rater agreement of results between VISA and specialist vision assessments.

Methods

The development and pilot validation of VISA have been described elsewhere (14). This study is reported in accordance with the STARD guidelines (15).

Design

A prospective case cohort comparative design was used for the validation clinical study between September 2016 and February 2019. Individuals were suitable for inclusion if they were 18 years of age or older, had clinical diagnosis of stroke as defined by World Health Organisation, had the ability to agree to vision screening using verbal or non-verbal indications of agreement, did not have severe cognitive impairment preventing screening and did not decline vision screening. This was a convenience sample of participants who were identified as being eligible from inpatients on the stroke unit. Our inclusion criteria were intended to be pragmatic and inclusive of as many stroke survivors as possible. The clinical study was undertaken in accordance with the Tenets of Helsinki with NHS research ethical approval. Research ethics approval was obtained separately for VISA print (16/NI/0125) and for VISA app (17/WA/0411). All participants provided informed consent.

Setting, Recruitment and Assessment

Recruitment took place across five hospitals (secondary hospital care) in which an orthoptist was a member of the core acute stroke unit multidisciplinary team (as per national guidelines: Royal College of Physicians Intercollegiate Stroke Guidelines and British & Irish Orthoptic Society extended guidelines for stroke practice) (16, 17).

For peer review only - http://bmjopen.bmj.com/site/about/guidelines.xhtml

For the purpose of this study, vision screening was undertaken with VISA and screening was defined as the assessment of stroke survivors for the presence of reduced visual function against pre-set abnormality criteria.

Specialist visual assessment was defined as the vision assessment undertaken by an orthoptist in which detection of visual impairment was coupled with formal diagnosis of the type of visual condition present. As a minimum this consisted of near and distance LogMAR visual acuity, cover test, ocular motility assessment, visual field to confrontation and visual inattention assessment.

Each stroke survivor underwent two vision assessments: the routine orthoptic specialist vision assessment and the VISA screening assessment.

VISA was available in print and as a software app. The app was approved by the Medicines and Healthcare products Regulatory Authority (MHRA: Reference CI/2017/0065) for NHS use in this study. There are five VISA sections comprising case history, LogMAR visual acuity at near and distance, eye alignment and movement, visual fields and visual inattention. A separate section comprising stand-alone user instructions is included. In brief, VISA consists of five sections. Section 1 comprises a case history with questions and observations of visual symptoms and signs. Section 2 comprises an assessment of LogMAR visual acuity for near and distance; monocular or binocular depending on the ability of the patient. Section 3 is an assessment of eye alignment and eye movements (smooth pursuits) into up, down, right and left gaze positions. Section 4 is an assessment of visual field, and section 5 is an assessment of visual inattention including line bisection, clock drawing and a cancellation task. The print and app versions are identical with the exception of the visual field assessment; in VISA app, a kinetic visual field assessment is undertaken which, at a test distance of 30cms and a screen width of 24.6cms, allows an assessment of the 40 degree visual field. The free-to-access VISA tool is available on; www.liverpool.ac.uk/psychology-health-and-society/departments/health-services-research/research/vision/ *(this link will go live on publication of this paper)*.

The routine orthoptic vision assessment comprised detailed diagnostic assessments of case history, visual acuity, ocular alignment and movement, visual field and visual perception. This assessment was undertaken within 24 hours (typically the same day) of the VISA screen – to minimise effect of potential recovery.

For peer review only - http://bmjopen.bmj.com/site/about/guidelines.xhtml

The order of the VISA screening and orthoptic vision assessments varied to avoid the effects of fatigue and bias towards either the screen or specialist assessment. The screener and orthoptist were blinded to each other's assessments to prevent bias of assessment. The within-assessment order of testing varied for the specialist assessment. However, the order of testing within the VISA screen followed a set order of 1) case history, 2) visual acuity, 3) eye alignment and movement, 4) visual field and 5) visual inattention assessments.

Statistical methodology and sample size

Results were taken in numerical format from the referral forms completed by both the screener and orthoptist. The specialist vision assessment was taken as the reference standard.

The primary outcome measure was a binary measure of the presence or absence of visual impairment (defined as reduced distance vision <0.2, reduced near vision <0.3 (equivalent to N6), eye movement abnormality, visual field loss, visual perceptual abnormality). The primary outcome measure was evaluated by Kappa values assessing chance-eliminated agreement between the results of the VISA screening and orthoptic vision assessment.

Secondary outcome measures were the calculation of sensitivity, specificity and predictive values. Level of sensitivity was estimated as the proportion of patients with visual impairment as diagnosed by the gold standard clinical examination, that are correctly identified by the screener, and the corresponding 95% confidence interval was calculated. Level of specificity was estimated as the proportion of patients without visual impairment that are correctly identified by the screener, and the corresponding 95% confidence interval. Further, we calculated the positive and negative predictive values for the VISA screen.

For sample size, we applied the principles for diagnostic accuracy studies, and aimed to recruit a sample of 100 for validation of VISA print and a further sample of 100 for VISA app (18).

Process evaluation

Process evaluation for acceptability of VISA during the clinical study was collected via clinician feedback sheets and one-to-one reports from patients. Feedback sheets could be returned at any time during the study to report any issues with testing alongside obtaining clinician views based on their use of VISA. Feedback sheets asked the following:

1. Are the instructions for the various tests clear?
2. Which instructions should be amended?
3. What additional instruction information/rewording do you suggest?

For peer review only - http://bmjopen.bmj.com/site/about/guidelines.xhtml

4. Which instructions require less information?
5. Are any tests not useful or difficulty to do? (Specify)
6. Should any other tests be added in?
7. How long does it take you to do the screen?
8. Other comments?

Comments collected from feedback sheets and reports were collated descriptively.

Patient and Public Involvement

Patients were involved in the design and monitoring of this study. Patients from the VISable stroke and vision panel were consulted when devising the study plan and conduct. Reports during the conduct of this study were circulated to the VISable panel for patient monitoring purposes.

Results

Completion rate

Two hundred and twenty-two stroke patients received both a VISA screening assessment and a reference vision assessment (during the period of September 2016 to February 2019).

All elements of the VISA screen were attempted by 201 patients. VISA print was used with 122 patients from which complete data was available for 101 for analysis. The mean age of patients on completion was 70.6 (SD 13.5), 46 were female and 54 male. The reported mean time of test duration was 23.5 minutes (SD 10.0).

VISA app was completed with 100 patients was a mean age of 63.4 (SD 13.4), of which 72 were male and 28 were female.

VISA print was fully completed by 91 patients, with the remaining 10 missing one or more elements (near vision n=5, distance vision n=5, ocular motility n=1, visual fields n=1, visual inattention n=9). The specialist vision assessment was fully completed by 90 patients, with the remaining 11 missing one or more elements (near vision n=8, visual inattention n=9). Reasons for inability to complete one or more of the elements were typically recorded as either cognitive impairment or fatigue. VISA app and specialist vision assessment were fully completed by all 100 patients. Missing data did not automatically result in failure for that section, thereby requiring referral. The reason for failure was taken into account; for example if a section was not completed due to fatigue this would not pragmatically have resulted in a referral but instead, a retest.

For peer review only - http://bmjopen.bmj.com/site/about/guidelines.xhtml

Referral agreement for VISA print

The agreement of whether to make a referral to specialist eye services based on the results of the VISA print versus those from specialist vision assessment had a Kappa value of 0.648 (substantial agreement) (95% CI 0.424 – 0.872).

Sensitivity of 97.67% and specificity of 60.00% were found. The positive and negative predictive values were 93.33% and 81.82% respectively. These calculations are outlined in Table 1. Agreement was found for 93 participants (nine had no visual impairment, 84 required referral because of failed screening) as outlined in Figure 1.

VISA print produced two false negative and six false positive results. Of the false negative results, both had ocular motility problems, of which one also had reduced near vision. The two ocular motility problems missed were asymptomatic minimal rotary nystagmus and limited elevation. The latter also had reduced near vision at 0.450 LogMAR. For false positive results, three with reduced near vison, two with ocular motility problems and one with both reduced near vision and visual inattention, were detected by screening and found not to be present by the orthoptic vision assessment. The referral relating to reduced near vision all detected N8 level of vision in one or both eyes. The referral relating to visual inattention were detected on the clock drawing element; it was noted by the examiner that the inaccurate completion was likely due to cognitive impairment. The ocular motility problems detected were reported as limitation of vertical gaze and nystagmus.

Test component agreement for VISA print

The agreements for the individual components between VISA print and specialist vision assessments are outlined in Table 2. The highest levels of agreement were produced for distance visual acuity (0.565) and visual fields (0.504), both with moderate agreement. The lowest level of agreement was produced for near visual acuity (0.236) and ocular motility (0.367), both with fair agreement. Low agreement for ocular motility related to high false positives and false negatives. In ten cases (one with multiple conditions) were not detected (false negative). These comprised of four defects of vertical movement (including one upgaze palsy, two restrictions of elevation and one V-pattern), three cases of nystagmus (including one minimal rotary nystagmus, one gaze-evoked and one end-point nystagmus), and four cases of reduced convergence. The low agreement with near visual acuity related to high false

For peer review only - http://bmjopen.bmj.com/site/about/guidelines.xhtml

negatives where 23 cases were not detected – these comprised of ten with 0.4 LogMAR or better, nine between 0.4 and 0.5 LogMAR and three 0.6 LogMAR or worse.

Referral agreement for VISA app

The agreement of whether to make a referral to specialist eye services based on the results of VISA app versus those from specialist vision assessment had a Kappa value of 0.690 (substantial agreement) (95% CI 0.528-0.851).

Sensitivity of 88.31% and specificity of 86.96% were calculated. The positive and negative predictive values were 95.77% and 68.97% respectively. These calculations are outlined in Table 3.

Agreement was found for 88 participants (20 had no visual impairment, 68 required referral because of failed screening) as outlined in Figure 1. VISA app produced nine false negative and three false positive results. Of the false negative results, four had slightly reduced near vision between 0.3 and 0.4 LogMAR, two had reduced distance vision of 0.3 LogMAR, two had mild visual inattention (one detected on clock cancellation and was the examiners judgement) and one had reduced near vision of 0.4 LogMAR and a visual field defect (partial right superior quadrantanopia detect on confrontation only, but not detected by formal perimetry using binocular Esterman). False positive results (one with reduced distance vision, one with a visual field defect and one with both a visual field defect and visual inattention) were detected by screening and found not to be present by the orthoptic vision assessment. The referral relating to reduce distance vision was 0.4 LogMAR. The referral relating to a visual field defect of general constriction and the visual inattention was detected on the longest line in the line bisection element. The other visual field defect detected was general constriction.

Test component agreement for VISA app

The agreements for the individual components between VISA app and orthoptic vision assessments are outlined in Table 4. The highest levels of agreement were produced for distance visual acuity (0.783) and visual fields (0.701), both with substantial agreement. The lowest level of agreement was produced for visual inattention (0.323) with fair agreement. The low agreement with visual inattention related to 16 false positives, of which 13 were detected with one of the three tests, 12 with line-bisection, 1 with clock drawing.

Perimetry agreement

For peer review only - http://bmjopen.bmj.com/site/about/guidelines.xhtml

Twenty-five participants had formal perimetry using the binocular Esterman programme rather than confrontation. There was perfect agreement (1.0) of whether a visual field defect was present between the kinetic visual field test on VISA app versus formal perimetry using the binocular Esterman programme. Twenty-one had a visual field defect and four were found to have a normal visual field.

Process evaluation

Information from feedback sheets and detailed notes from interviews were compiled and grouped for type of feedback. Minimal feedback was obtained during the validation study. Feedback related to the duration of screening, presentation of tests on the app and referral guides. One stroke unit noted that VISA could take too long in the hyper-acute stage with unwell patients. Feedback on app presentation included a change to the clock drawing circle (to remove lines that might indicate time markers), change to the fixation target for the visual field test, additional of a nystagmus check on eye movement testing (in addition to its presence in the case history checklist) and ability to delete erroneous marks on the line bisection test. For referral guidance, feedback requested the addition of a refer / retest icon on the patient results page. Further feedback reported greater ease of screening with the app for those having to use their non-dominant hand because of upper limb motor impairment. Stroke survivors found it easier to respond using the touch screen than traditional pen and paper tasks when using their non-dominant hand.

Discussion

In this study, we present the VISA screening tool, performed by non-eye trained specialists, with validation results for the printed version and for the software app. Overall, referral had sensitivity and specificity of >88% and >60% respectively, positive and negative predictive values of >93% and >68% respectively, with substantial agreement between VISA screening and comprehensive orthoptic assessment of about Kappa 0.7. Agreement was lowest for eye movement screening, near visual acuity and visual inattention whereas all other individual sections showed higher levels of agreement. Process evaluation aided further refinement of VISA and, in particular, changes to presentation features on the app version.

When designing and using screening tools there is a balance between sensitivity and specificity for reliable detection of deficits. Low agreement in the VISA sections related to high

For peer review only - http://bmjopen.bmj.com/site/about/guidelines.xhtml

false positive referrals where VISA screen indicated a fail for ocular motility or visual inattention. The specialist vision assessment confirmed ocular motility changes which were classed as 'normal' physiological eye movement patterns such age-related reduced elevation, and which alone would not have required referral. False positive referrals for visual inattention occurred where the patient failed to complete the section because of fatigue or cognitive impairment. Reduced visual acuity was always at a borderline level just above the fail threshold.

False negative referrals are important to consider; failed detection of significant deficits is to be avoided. Our results showed low numbers of false negatives which included failed detection of ocular motility defects, reduced visual acuity, visual inattention and visual field defect. The ocular motility defects were related to asymptomatic limited elevation and minimal nystagmus which would not have constituted referrals by orthoptic vision assessment. Reduced visual acuity, similar to the false positive results, was always close to the pass/fail threshold. Arguably, this is an ideal call for retest rather than refer. One case of mild visual inattention was not passed by VISA app where the diagnosis had been made by clinical observation. One visual field defect related to a peripheral field loss; a defect that could not be detected by the central testing area of VISA app.

Specificity was higher when using VISA app compared to VISA print. This is likely due to the staff mix using VISA. VISA print was used solely by members of the stroke team and often without any formal vision training. VISA app was used by a mix of stroke team members but also orthoptists. Accuracy was likely enhanced by involvement of the latter.

VISA print and app provide a vision screen across the main categories of potential visual impairment following stroke. Besides a case history section, screening includes visual acuity, eye position and movements, visual fields and visual inattention. There are potential advantages for using either the manual tool or the app. Some clinicians and stroke survivors may prefer and respond better to use of traditional testing options inclusive of pen and paper tasks. The recording charts are completed during the testing period and can be entered in hospital case notes immediately. The app produces a PDF file of results which has to be printed before entry in hospital case notes. Conversely, the PDF file is an advantage for electronic hospital records. Further, the app provides a constant background illumination for screening assessments whereas the manual is used under variable lighting conditions dependent on wherever the screening is undertaken. The app uses a kinetic central visual

For peer review only - http://bmjopen.bmj.com/site/about/guidelines.xhtml

field assessment that is run as a standardised test which reduces examiner bias – a bias that persists for confrontation visual field assessment.

A systematic review of screening options for post-stroke visual impairment reported vision screening checklists and stroke screening tools (e.g. National Institute for Health Stroke Scale) which include elements of vision assessment, however not all potential visual impairments are screened (19). Past vision screening publications have reported the results of vision 'checklists' – lists of information gathered from questioning the patient, observations or from data documented in the case notes. The Vision In Stroke (VIS) reported checklist screening in 915 stroke survivors with sensitivity of 0.42, specificity of 0.52 and agreement against a reference standard of 0.428 (-0.048 to 0.019 CI: Kappa) (2, 12). An Australian study reported the use of a checklist for detection of eye conditions and vision defects in 100 stroke survivors with 69% accuracy and intra-class correlation of 0.84 (0.77-0.89 CI) (20). More recently, a vision screening app (available on android platforms) was developed for use with stroke survivors (StrokeVision app) with sections assessing visual acuity, visual fields and visual inattention (21). This was validated with a cohort of 45 stroke survivors with sensitivities across the various sections of 50-79% and specificities of 87-98%. The specificities reported are higher than those from our VISA study but likely reflect the use of the StrokeVision app by fully trained research assistants versus the VISA completion by members of the stroke multidisciplinary team who only followed the in-built screening instructions.

Overall, vision tools/apps provide a more extensive screening of vision with greater accuracy than vision checklists. However, there are issues with how best to identify the presence of visual impairment through stroke team vision screening and specialist vision assessment (19, 22). Even with screening measures in place there are also issues reported with provision of care and access to vision services for stroke survivors who have been identified as having vision problems (13).

An ideal stroke vision service follows recommendations from the National Clinical Guidelines for Stroke which specify orthoptists as core members of the acute stroke team and screen all stroke survivors prior to discharge (16). Despite consistent findings that inclusion of vision services within the multi-disciplinary team (MDT) is highly beneficial, such visual assessment is not common and services are inconsistent throughout the UK. Stepped models of care must be considered to meet the needs of stroke survivors against the context of local service capacity. Access to orthoptic services on acute stroke units enables faster provision of vision

For peer review only - http://bmjopen.bmj.com/site/about/guidelines.xhtml

screening. The earlier assessment time-point reported for the IVIS study is important as it shows the feasibility and acceptability of early visual assessment within 3 days of stroke onset for at least half of stroke survivors and within 1 week of stroke onset for the majority (4). This in turn allows early detection of visual impairment and sharing of the functional significance of this with the patients, carers and stroke teams. Furthermore, early assessment leads to early intervention which has potential impact on general rehabilitation where visual function can be improved (2, 3, 10). In the absence of orthoptic services, further stepped down models of care include the use of screening tools or screening checklists.

Such screening methods cannot replace the accuracy of a reference, specialist vision assessment. However, they serve an important purpose of obtaining a standardised screen in the absence of on-site specialist vision services and are better than no or non-standardised assessments by non-eye trained clinicians. In such instances, we advise the use of a screening tool. The advantages of VISA print and app are their validation in a real-world pragmatic study conducted in acute stroke units and used by non-eye trained clinicians. Clinicians used the in-built standalone instructions – designed to avoid the need of regular specialist vision training which can be difficult to access or provide. Further, the app is MHRA approved for clinical assessment; an important requirement for NHS adoption. The availability of VISA as a manual and as an app facilitates use alongside paper-based records or integration with electronic patient record systems.

Vision checklists have been shown to have a low sensitivity and specificity, and an over-reliance on the report of visual symptoms (2, 12). The VISA print and app offer an intermediate measure between vision checklists and orthoptic specialist vision services with greater accuracy than vision checklists but lacking the accuracy of orthoptic assessments and the immediate access to management of visual problems provided by orthoptic stroke unit services. The VISA print/app does not preclude the use of vision checklists however. For some stroke survivors who are very unwell acutely and/or lack sufficient cognition and communication, simpler vision checklists are quick and easy to use in such circumstances and remain more accurate than no vision screen at all.

There are some limitations to consider for this study. The VISA screening tool was used on acute stroke units. There was no validation of the tool in community settings. However, there

For peer review only - http://bmjopen.bmj.com/site/about/guidelines.xhtml

is little reason to think it would be of less or use or accuracy when used in other stroke settings. Test-retest variability of the VISA screening tool was not evaluated during this study as this would have caused too high a burden of assessment on participants.

Conclusions

Validation of the VISA screening tool in either print or app format shows improved detection accuracy for detection of stroke-related visual impairment by clinicians involved in stroke care who are not specialists in vision problems and lack formal eye training. Where early visual impairment detection occurs, this facilitates prompt referral with fewer false positives and negatives. Clinicians reported acceptability of the VISA screening tool for is use in screening for presence of vision problems in stroke survivors. Referral sensitivity of >88% and specificity of >60% were found for the VISA screening with substantial inter-rater agreement for referral between VISA screening and specialist vision assessments. The VISA screening tool provides a standardised and validated method to screen for visual problems following stroke and may further be of potential use for visual screening in other care settings such as neuro-rehabilitation.

Acknowledgements

We thank the VISable panel for their involvement in the design and monitoring of this study. We thank the patients and staff at Arrowe Park Hospital, Bradford Teaching Hospital, Manchester University Hospital, Salford Royal Hospital and the Walton Centre; specifically Felicity Hale, Jennifer Lawrence, Sam Campbell, Claire Fowler, Imtiaz Nazir, Laura Stubbs, Abigail Kerr, Stephanie Wolff, Kim Cochrane, Tamsin Steward, Ella Henry, Carmel Noonan and Hayley Draper.

References

1. Hepworth LR, Rowe FJ, Walker MF, et al. Post-stroke visual impairment: a systematic literature review of types and recovery of visual conditions. Ophthalmology Research. 2016;5(1).
2. Rowe F, VIS writing group. Vision In Stroke cohort: profile overview of visual impairment. Brain and Behavior. 2017.
3. Jones SA, Shinton RA. Improving outcome in stroke patients with visual problems. Age and Ageing. 2006;35(6):560-5.
4. Rowe FJ, Hepworth LR, Howard C, et al. High incidence and prevalence of visual problems after acute stroke: an epidemiology study with implications for service delivery. PLoS One. 2019;14(3).

For peer review only - http://bmjopen.bmj.com/site/about/guidelines.xhtml

5. Stroke Association. State of the nation: stroke statistics 2018 [Available from: https://www.stroke.org.uk/system/files/sotn_2018.pdf.
6. Hepworth LR, Rowe FJ. Visual impairment following stroke - the impact on quality of life: a systematic review. Ophthalmology Research. 2016;5(2):1-15.
7. Tsai S-Y, Cheng C-Y, Hsu W-M, et al. Association between visual impairment and depression in the elderly. Journal of Formosan Medical Association. 2003;102(2):86-90.
8. Chen CS, Lee AW, Clarke G, et al. Vision-related quality of life in patients with complete homonymous hemianopia post stroke. Topics in Stroke Rehabilitation. 2009;16(6):445-53.
9. Gall C, Lucklum J, Sabel BA, et al. Vision- and health-related quality of life in patients with visual field loss after postchiasmatic lesions. Investigative Ophthalmology and Vision Science. 2009;50(6):2765-76.
10. Hanna KL, Hepworth LR, Rowe FJ. The treatment methods for post-stroke visual impairment: a systematic review. Brain and Behavior. 2017;7(5).
11. Rowe F, Walker M, Rockliffe J, et al. Delivery of high quality stroke and vision care: experiences of UK services. Disability and Rehabilitation. 2016;38(8):813-7.
12. Rowe FJ, VIS Group UK. Accuracy of referrals for visual assessment in a stroke population. Eye. 2011;25(2):161-7.
13. Rowe FJ, Walker M, Rockliffe J, et al. Care provision for poststroke visual impairment. Journal of Stroke and Cerebrovascular Diseases. 2015;24(6):1131-44.
14. Rowe FJ, Hepworth LR, Hanna KL, et al. Visual Impairment Screening Assessment (VISA) tool: pilot validation. BMJ Open. 2018.
15. Cohen JF, Korevaar DA, Altman DG, et al. STARD 2015 guidelines for reporting diagnostic accuracy studies: explanation and elaboration. BMJ Open. 2016;6(e012799).
16. Intercollegiate Stroke Working Party. National clinical guideline for stroke. 5th ed. London: Royal College of Physicians; 2016.
17. British and Irish Orthoptic Society. Compentency standards and professional practice guidelines: extended practioner role London: British and Irish Orthoptic Society,; 2018.
18. Bland JM, Altman DG. Statistical methods for assessing agreement between two methods of clinical measurement. Lancet. 1986;327(8476):307-10.
19. Hanna KL, Hepworth LR, Rowe FJ. Screening methods for post-stroke visual impairment: a systematic review. Disability and Rehabilitation. 2017;39(25):2531-43.
20. Jolly N, Macfarlane A, Heard R. Towards gaining the best information about vision to assist the recovery of a patient with stroke. Strabismus. 2013;21(2):145-9.
21. Quinn TJ, Livingstone I, Weir A, et al. Accuracy and feasibility of an android-based digital assessment tool for post stroke visual disorders - the StrokeVision app. Front Neurology. 2018;9(146).
22. Rowe FJ. Who sees visual impairment following stroke? Strabismus. 2010;18(2):37-40.

For peer review only - http://bmjopen.bmj.com/site/about/guidelines.xhtml

Table 1: Calculations of sensitivity, specificity and predictive values for VISA print

Positive	
True positive, i.e. visual impairment present and referred	84
False negative, i.e. visual impairment present but not referred	2
Negative	
False positive, i.e. visual impairment not present but referred	6
True negative, i.e. visual impairment not present and not referred	9
Output	
Sensitivity (true positive/true positive + false negative)	97.67% (95% CI: 91.85 – 99.72%)
Specificity (true negative/false positive + true negative)	60.00% (95% CI: 32.29 – 83.66%)
Positive predictive value (true positive/false positive + true positive)	93.33% (95% CI: 88.27 – 96.30%)
Negative predictive value (true negative/false negative + true negative)	81.82% (95% CI: 51.83 – 94.95%)

For peer review only

Table 2: Summary of agreement between VISA print and specialist vision assessment for referral to specialist eye services and individual components

Element of testing	Agreement	False negative	False positive	Kappa value (95% CI)
Referral	93	2	6	0.648 (0.424 – 0.872)
Near visual acuity	65	23	12	0.236 (0.045 – 0.427)
Distance visual acuity	79	9	13	0.565 (0.405 – 0.725)
Ocular alignment	89	5	7	0.388 (0.110 – 0.667)
Ocular motility	72	10	19	0.367 (0.181 – 0.553)
Visual fields	76	7	18	0.504 (0.339 – 0.668)
Visual inattention	74	4	21	0.500 (0.340 – 0.659)

For peer review only

Table 3: Calculations of sensitivity, specificity and predictive values for VISA app

Positive	
True positive, i.e. visual impairment present and referred	68
False negative, i.e. visual impairment present but not referred	9
Negative	
False positive, i.e. visual impairment not present but referred	3
True negative, i.e. visual impairment not present and not referred	20
Output	
Sensitivity (true positive/true positive + false negative)	88.31%<br>(95% CI: 78.97 – 94.51%)
Specificity (true negative/false positive + true negative)	86.96%<br>(95%CI: 66.41 – 97.22%)
Positive predictive value (true positive/false positive + true positive)	95.77%<br>(95% CI: 88.72 – 98.49%)
Negative predictive value (true negative/false negative + true negative)	68.97%<br>(95% CI: 54.10 – 80.73%)

For peer review only

Table 4: Summary of agreement between VISA app and specialist vision assessment for referral to specialist eye services and individual components.

**Element of testing**	**Agreement**	**False negative**	**False positive**	**Kappa value (95% CI)**
Referral	88	9	3	0.690 (0.528 – 0.851)
Near visual acuity	77	19	3	0.416 (0.227 – 0.605)
Distance visual acuity	90	6	4	0.783 (0.656 – 0.910)
Visual fields	85	3	12	0.701 (0.564 – 0.838)
Visual inattention	78	6	16	0.323 (0.108 – 0.538)

For peer review only

Figure 1: Flow diagram of participant outcome for VISA screening and orthoptic full assessment.

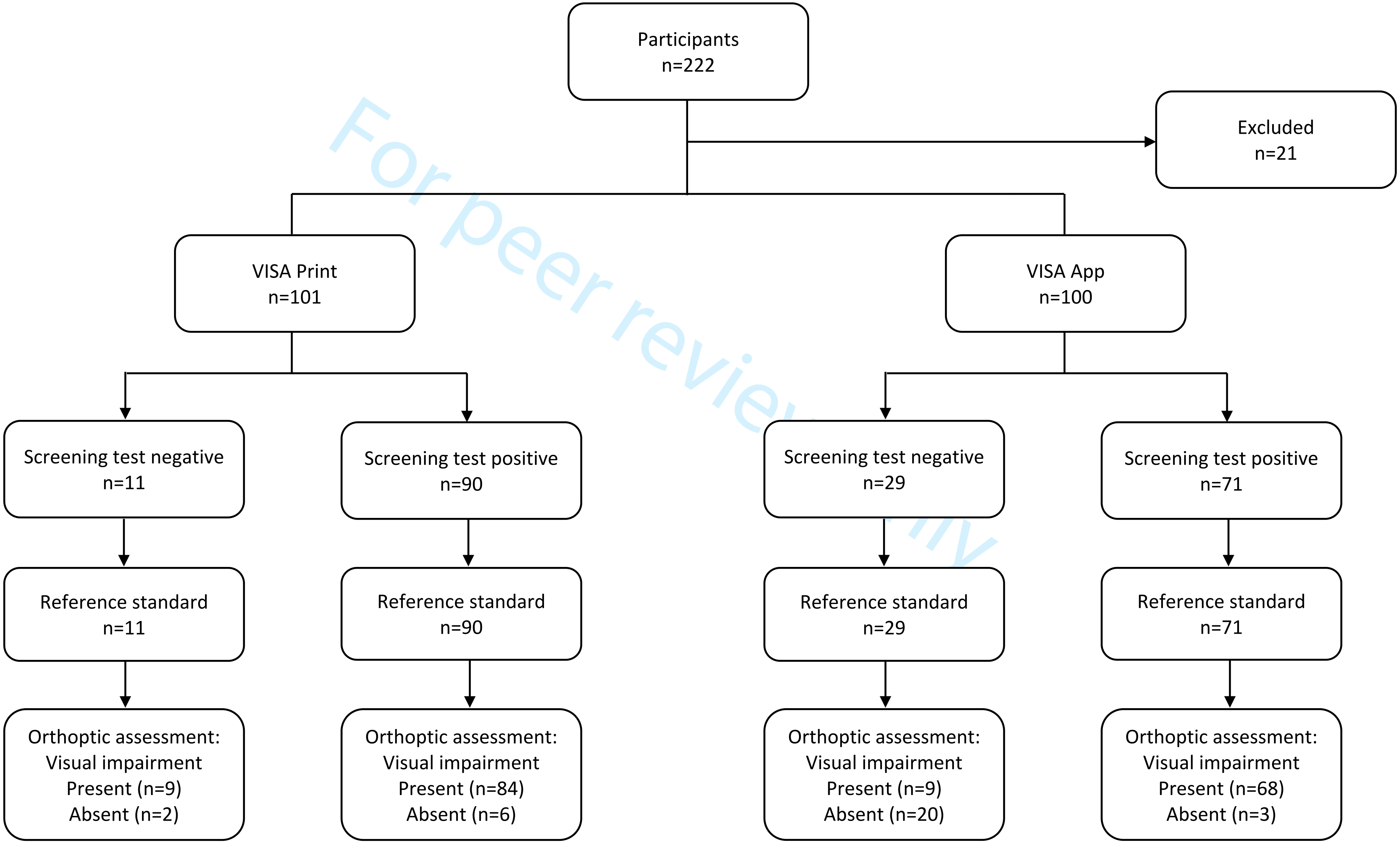

1 Section & Topic	No	Item	Reported on page #
**TITLE OR ABSTRACT**			
	**1**	Identification as a study of diagnostic accuracy using at least one measure of accuracy (such as sensitivity, specificity, predictive values, or AUC)	1
**ABSTRACT**			
	**2**	Structured summary of study design, methods, results, and conclusions (for specific guidance, see STARD for Abstracts)	2
**INTRODUCTION**			
	**3**	Scientific and clinical background, including the intended use and clinical role of the index test	3
	**4**	Study objectives and hypotheses	3
**METHODS**			
Study design	**5**	Whether data collection was planned before the index test and reference standard were performed (prospective study) or after (retrospective study)	3
Participants	**6**	Eligibility criteria	3-4
	**7**	On what basis potentially eligible participants were identified (such as symptoms, results from previous tests, inclusion in registry)	4
	**8**	Where and when potentially eligible participants were identified (setting, location and dates)	3-4
	**9**	Whether participants formed a consecutive, random or convenience series	4
Test methods	**10a**	Index test, in sufficient detail to allow replication	4
	**10b**	Reference standard, in sufficient detail to allow replication	4
	**11**	Rationale for choosing the reference standard (if alternatives exist)	N/A
	**12a**	Definition of and rationale for test positivity cut-offs or result categories of the index test, distinguishing pre-specified from exploratory	3 – in previously published paper
	**12b**	Definition of and rationale for test positivity cut-offs or result categories of the reference standard, distinguishing pre-specified from exploratory	5
	**13a**	Whether clinical information and reference standard results were available to the performers/readers of the index test	5
	**13b**	Whether clinical information and index test results were available to the assessors of the reference standard	5
Analysis	**14**	Methods for estimating or comparing measures of diagnostic accuracy	5
	**15**	How indeterminate index test or reference standard results were handled	6
	**16**	How missing data on the index test and reference standard were handled	6
	**17**	Any analyses of variability in diagnostic accuracy, distinguishing pre-specified from exploratory	N/A
	**18**	Intended sample size and how it was determined	5
**RESULTS**			
Participants	**19**	Flow of participants, using a diagram	Figure 1
	**20**	Baseline demographic and clinical characteristics of participants	6
	**21a**	Distribution of severity of disease in those with the target condition	7-8
	**21b**	Distribution of alternative diagnoses in those without the target condition	N/A
	**22**	Time interval and any clinical interventions between index test and reference standard	5
Test results	**23**	Cross tabulation of the index test results (or their distribution) by the results of the reference standard	Table 1 and 3
	**24**	Estimates of diagnostic accuracy and their precision (such as 95% confidence intervals)	6-7 and Tables 1 and 3
	**25**	Any adverse events from performing the index test or the reference standard	9
**DISCUSSION**			
	**26**	Study limitations, including sources of potential bias, statistical uncertainty, and generalisability	12
	**27**	Implications for practice, including the intended use and clinical role of the index test	11-12
**OTHER INFORMATION**			
	**28**	Registration number and name of registry	N/A
	**29**	Where the full study protocol can be accessed	N/A
	**30**	Sources of funding and other support; role of funders	1

For peer review only

For peer review only

BMJ Open

The Vision Screening Assessment (VISA) tool - diagnostic accuracy validation of a novel screening tool in detecting visual impairment among stroke survivors.

Journal:	*BMJ Open*
Manuscript ID	bmjopen-2019-033639.R1
Article Type:	Original research
Date Submitted by the Author:	12-Dec-2019
Complete List of Authors:	Rowe, Fiona; University of Liverpool, Health Services Research<br>Hepworth, Lauren; University of Liverpool, Health Services Research<br>Howard, Claire; University of Liverpool, Health Services Research<br>Bruce, Alison; Bradford Institute for Health Research, ; University of York, Health Sciences<br>Smerdon, Victoria; Arrowe Park Hospital, Orthoptics<br>Payne, Terry; University of Liverpool, Computer Science<br>Jimmieson, Phil; University of Liverpool, Computer Science<br>Burnside, Girvan; University of Liverpool Faculty of Health and Life Sciences, Biostatistics
**Primary Subject Heading**:	Cardiovascular medicine
Secondary Subject Heading:	Ophthalmology
Keywords:	VISA, Screening, STROKE MEDICINE, Visual impairment, Orthoptics, Detection

SCHOLARONE™
Manuscripts

I, the Submitting Author has the right to grant and does grant on behalf of all authors of the Work (as defined in the below author licence), an exclusive licence and/or a non-exclusive licence for contributions from authors who are: i) UK Crown employees; ii) where BMJ has agreed a CC-BY licence shall apply, and/or iii) in accordance with the terms applicable for US Federal Government officers or employees acting as part of their official duties; on a worldwide, perpetual, irrevocable, royalty-free basis to BMJ Publishing Group Ltd ("BMJ") its licensees and where the relevant Journal is co-owned by BMJ to the co-owners of the Journal, to publish the Work in this journal and any other BMJ products and to exploit all rights, as set out in our licence.

The Submitting Author accepts and understands that any supply made under these terms is made by BMJ to the Submitting Author unless you are acting as an employee on behalf of your employer or a postgraduate student of an affiliated institution which is paying any applicable article publishing charge ("APC") for Open Access articles. Where the Submitting Author wishes to make the Work available on an Open Access basis (and intends to pay the relevant APC), the terms of reuse of such Open Access shall be governed by a Creative Commons licence – details of these licences and which Creative Commons licence will apply to this Work are set out in our licence referred to above.

Other than as permitted in any relevant BMJ Author's Self Archiving Policies, I confirm this Work has not been accepted for publication elsewhere, is not being considered for publication elsewhere and does not duplicate material already published. I confirm all authors consent to publication of this Work and authorise the granting of this licence.

For peer review only

The Vision Screening Assessment (VISA) tool - diagnostic accuracy validation of a novel screening tool in detecting visual impairment among stroke survivors.

Fiona J Rowe[1,2,3], Lauren R Hepworth[1,3,4], Claire Howard[1,3], Alison Bruce[4], Victoria Smerdon[5], Terry Payne[6], Phil Jimmieson[6], Girvan Burnside[7]

1, Department of Health Services Research, University of Liverpool, UK

2, Department of Ophthalmology, Walton Centre for Neurology and Neurosurgery, Liverpool, UK

3, Department of Orthoptics, Salford Royal NHS Foundation Trust, Salford, UK

4, Bradford Institute for Health Research, Bradford Teaching Hospitals NHS Foundation Trust, Bradford, UK

5, Department of Orthoptics, Wirral University Teaching Hospital NHS Foundation Trust, Liverpool, UK

6, Department of Computer Science, University of Liverpool, UK

7, Department of Biostatistics, University of Liverpool, UK

Address for correspondence:

Prof Fiona Rowe

Department of Health Services Research

Waterhouse Building Block B, 2nd floor,

University of Liverpool,

1-5 Brownlow Street,

Liverpool L69 3GL

E: rowef@liverpool.ac.uk

T: 0151 7944956

Disclosures: The authors report no conflict of interest for this study.

Funding: This study was supported by the University of Liverpool.

Word count: 4187

Cover title: Visual impairment screening assessment tool (VISA)

Number of figures: 1

For peer review only - http://bmjopen.bmj.com/site/about/guidelines.xhtml

Number of tables: 4

Keywords: VISA; Screening; Stroke; Visual impairment; Orthoptics; Detection

Author contributions: FR provided oversight for the study and led the writing of the paper. FR, LH, CH, VS and AB contributed to data collection, reviewing the draft paper and approving the final version. TP and PJ contributed to the development of VISA app software and its testing, and reviewed the draft paper and approved the final version. GB contributed to the statistical analysis and interpretation, reviewed draft amendments and approved the final version.

Data access; Data can be accessed via direct contact with the lead author.

For peer review only

Abstract

Purpose: Screening for visual problems in stroke survivors is not standardised. Visual problems that remain undetected or poorly identified can create unmet needs for stroke survivors. We report the validation of a new Vision Impairment Screening Assessment (VISA) tool intended for use by the stroke team to improve identification of visual impairment in stroke survivors.

Methods: We conducted a prospective case cohort comparative study in four centres to validate the VISA tool against a specialist reference vision assessment. VISA is available in print or as an app (MHRA regulatory approved); these were used equally for two groups. Both VISA and the comprehensive reference vision assessment measured case history, visual acuity, eye alignment, eye movements, visual field and visual inattention. The primary outcome measure was the presence or absence of visual impairment.

Results: Two hundred and twenty-one stroke survivors were screened. Specialist reference vision assessment was by experienced orthoptists. Full completion of screening and reference vision assessment was achieved for 201 stroke survivors. VISA print was completed for 101 stroke survivors; VISA app was completed for 100. Sensitivity and specificity of VISA print was 97.67% and 66.67% respectively. Overall agreement was substantial; K=0.648. Sensitivity and specificity of VISA app was 88.31% and 86.96% respectively. Overall agreement was substantial; K=0.690. Lowest agreement was found for screening of eye movement and near visual acuity.

Conclusions: This validation study indicates acceptability of VISA for screening of potential visual impairment in stroke survivors. Sensitivity and specificity were high indicating the accuracy of this screening tool. VISA is available in print or as an app allowing versatile uptake across multiple stroke settings.

Strengths and limitations of this study

- Validation of the VISA screening tool in this prospective study shows improved detection accuracy for detection of stroke-related visual impairment
- The study included clinicians involved in stroke care who are not specialists in vision problems and lack formal eye training.
- Where early visual impairment detection occurs, this facilitates prompt referral with fewer false positives and negatives.
- Through process evaluation, clinicians reported acceptability of the VISA screening tool for is use in screening for presence of vision problems in stroke survivors.
- The VISA screening tool may further be of potential use for visual screening in other care settings such as neuro-rehabilitation.

Introduction

The prevalence of overall visual impairment has been estimated at 65-73% with varying prevalence reported for specific types of visual impairment (inclusive of reduced central vision, ocular motility defects, visual field loss and visual perception problems) [1-4]. Figures for the incidence of new onset visual impairment following stroke are placed at about 60% [4]. Given the estimated 100,000 new onset strokes per annum in the UK there are sizeable numbers of stroke survivors living with stroke-related visual impairment [5].

Visual impairment constitutes a considerable comorbidity of stroke. Visual impairment, on its own or in addition to other stroke-related disabilities, can cause significant impact to quality of life [6]. For many, it results in inability or altered ability to undertake many aspects of daily activities with impact on return to work, participation in hobbies and family life, and can lead to social isolation, altered mood and depression [7-9]. Interventions for stroke-related visual impairment are well established [10] but require referral to appropriate eye care services, which is facilitated through orthoptic service routes [11]. Where visual

For peer review only - http://bmjopen.bmj.com/site/about/guidelines.xhtml

impairment is identified, this facilitates optimisation of other therapy and early access to vision rehabilitation.

There are issues with how best to identify the presence of visual impairment through stroke team vision screening and specialist vision assessment [12]. Even with screening measures in place there are also issues reported with provision of care and access to vision services for stroke survivors who have been identified as having vision problems [13].

The overall aim of this study was to validate the Vision Impairment Screening Assessment (VISA) tool which uses simple established assessments of visual function coupled with detailed instructions. Our objectives were to test VISA, available in print or as a software application, against a reference of a specialist vision assessment to determine sensitivity, specificity, predictive values and inter-rater agreement of results between VISA and specialist vision assessments.

Methods

The development and pilot validation of VISA have been described elsewhere [14]. This study is reported in accordance with the STARD guidelines [15].

Design

A prospective case cohort comparative design was used for the validation clinical study between September 2016 and February 2019. Individuals were suitable for inclusion if they were 18 years of age or older, had clinical diagnosis of stroke as defined by World Health Organisation, had the ability to agree to vision screening using verbal or non-verbal indications of agreement, did not have severe cognitive impairment preventing screening defined as difficulty with memory/concentration/decision making and thus being unable to follow instructions, and did not decline vision screening. This was a convenience sample of participants who were identified as being eligible from inpatients on the acute stroke unit. With recruitment on the acute stroke unit, time to VISA assessment was typically within one week of stroke onset. Our inclusion criteria were intended to be pragmatic and inclusive of as many stroke survivors as possible. The clinical study was undertaken in accordance with the Tenets of Helsinki with NHS research ethical approval. Research ethics approval was obtained separately for VISA print (16/NI/0125) and for VISA app (17/WA/0411). All participants provided informed consent.

Setting, Recruitment and Assessment

For peer review only - http://bmjopen.bmj.com/site/about/guidelines.xhtml

Recruitment took place across five hospitals (secondary hospital care) in which an orthoptist was a member of the core acute stroke unit multidisciplinary team (as per national guidelines: Royal College of Physicians Intercollegiate Stroke Guidelines and British & Irish Orthoptic Society extended guidelines for stroke practice) [16, 17].

For the purpose of this study, vision screening was undertaken with VISA and screening was defined as the assessment of stroke survivors for the presence of reduced visual function against pre-set abnormality criteria, outlined in the statistical methodology section.

Specialist visual assessment was defined as the vision assessment undertaken by an orthoptist in which detection of visual impairment was coupled with formal diagnosis of the type of visual condition present. As a minimum this consisted of near and distance LogMAR visual acuity, cover test, ocular motility assessment, standardised visual field to confrontation using 10mm red targets and visual inattention assessment.

Each stroke survivor underwent two vision assessments: the routine orthoptic specialist vision assessment and the VISA screening assessment. Patients were recruited consecutively as being identified to meet the inclusion criteria and providing consent to participate.

VISA was available in print and as a software app. The app was approved by the Medicines and Healthcare products Regulatory Authority (MHRA: Reference CI/2017/0065) for NHS use in this study. VISA was used in print form for the first half of recruitment and, subsequently in app form for the second half of recruitment to this study. Both VISA formats consisted of five VISA sections comprising case history, LogMAR visual acuity at near and distance, eye alignment and movement, visual fields and visual inattention. A separate section comprising stand-alone user instructions is included. In brief, VISA consists of five sections. Section 1 comprises a case history with questions and observations of visual symptoms and signs. When it is not possible to obtain a case history from the patient, the tool advises to consult family members/carers. The person completing the screen is instructed to observe for abnormalities of lids, pupils and head position among other vision signs. Section 2 comprises an assessment of LogMAR visual acuity for near (35cm) and distance (3 metres); monocular or binocular depending on the ability of the patient. A matching card was available for patients who were unable to name letters but could point to letters. For those unable to comply with any letter test, a further option included grating cards that utilise a preferential looking technique which is particularly useful with cognitive/communication issues. Section 3 is an assessment of eye alignment observing

For peer review only - http://bmjopen.bmj.com/site/about/guidelines.xhtml

symmetry of the corneal reflections of each eye. Clinician observations can be compared to images of straight eyes or images of eyes in converged, diverged, elevated or depressed strabismus positions. Eye movements (smooth pursuits) assessed full movements of each eye into up, down, right and left gaze positions. Clinician observations could be compared to images of full ocular rotations to right/left gaze, elevation/depression and on convergence. Section 4 is an assessment of visual field, and section 5 is an assessment of visual inattention including line bisection, clock drawing and a cancellation task. The print and app versions are identical with the exception of the visual field assessment. In VISA print, a standardised method of confrontation is conducted. Confrontation follows a typical method with the clinician seated directly opposite the patient at a distance of 1metre and following stages that involve the patient indicating when a 10mm red target is seen in the periphery of their vision, finger counting in each quadrant of the visual field and comparison of examiner facial features. In VISA app, a kinetic visual field assessment is undertaken which, at a test distance of 30cms and a screen width of 24.6cms, allows an assessment of the 40 degree visual field. The patient is asked to fixate a static fixation point in the corner of the screen whilst a stimulus moves from the other edges. They are asked to tap the tablet screen when the stimulus is seen. This is repeated with the fixation target positioned at all four corners of the screen.

Section 5 includes three routine assessments for visual inattention; line bisection, clock drawing and a cancellation task. The line bisection task requires the patient to indicate the centre of line for three lines of differing lengths. The cancellation task requires the patient to cross out large clock symbols amongst distractors of small clock symbols and large/small open circles. Clock drawing requires the patient to draw the numbers and clock hands on a blank circle. VISA app collates data from each of the sections to create a pdf record of the assessment. The free-to-access VISA tool is available on; www.vision-research.co.uk *(this link will go live on publication of this paper)*.

The routine orthoptic vision assessment comprised detailed diagnostic assessments of case history, visual acuity, ocular alignment and movement, visual field and visual perception. This assessment was undertaken within 24 hours (typically the same day) of the VISA screen – to minimise effect of potential recovery. The orthoptic assessment covered all assessment sections included in the VISA tool. However the orthoptist undertook a detailed assessment

For peer review only - http://bmjopen.bmj.com/site/about/guidelines.xhtml

using their specialist expertise to interpret the results and adapt testing methods to individual requirements.

The order of the VISA screening and orthoptic vision assessments varied in a pragmatic manner to avoid the effects of fatigue and bias towards either the screen or orthoptic vision assessment. The screener and orthoptist were blinded to each other's assessments to prevent bias of assessment. The within-assessment order of testing varied for the orthoptic assessment. However, the order of testing within the VISA screen followed a set order of 1) case history, 2) visual acuity, 3) eye alignment and movement, 4) visual field and 5) visual inattention assessments.

Statistical methodology and sample size

Results were taken in numerical format from the referral forms completed by both the screener and orthoptist. The orthoptic vision assessment was taken as the reference standard.

The primary outcome measure was a binary measure of the presence or absence of visual impairment (defined as one or more of the following; reduced distance vision <0.2, reduced near vision <0.3 (equivalent to N6), deviated eye position, eye movement abnormality (incomplete eye rotations in any position of gaze), visual field loss (e.g. presence of hemianopia, quadrantanopia, constriction), visual inattention with displaced line bisection, <42 score on cancellation task and/or incomplete/displaced clock drawing). The primary outcome measure was evaluated by Kappa values assessing chance-eliminated agreement between the results of the VISA screening and orthoptic vision assessment.

Secondary outcome measures were the calculation of sensitivity, specificity and predictive values. Level of sensitivity was estimated as the proportion of patients with visual impairment as diagnosed by the gold standard clinical examination, that are correctly identified by the screener, and the corresponding 95% confidence interval was calculated. Level of specificity was estimated as the proportion of patients without visual impairment that are correctly identified by the screener, and the corresponding 95% confidence interval. Further, we calculated the positive and negative predictive values for the VISA screen. Kappa (K) values assessed chance-eliminated agreement between the individual components of VISA tool and orthoptic vision assessment. The interpretation used was 0.0-0.2 poor, 0.21-0.4 fair, 0.41-0.6 moderate, 0.61-0.8 substantial and 0.81-1.0 almost perfect [18]. Analysis was conducted using StatsDirect software (StatsDirect Ltd).

For peer review only - http://bmjopen.bmj.com/site/about/guidelines.xhtml

For sample size, we applied the principles for diagnostic accuracy studies, and aimed to recruit a sample of 100 for validation of VISA print and a further sample of 100 for VISA app [19].

Process evaluation

Process evaluation for acceptability of VISA during the clinical study was collected via clinician feedback sheets and one-to-one reports from patients. Feedback sheets could be returned at any time during the study to report any issues with testing alongside obtaining clinician views based on their use of VISA. Feedback sheets asked the following:

1. Are the instructions for the various tests clear?
2. Which instructions should be amended?
3. What additional instruction information/rewording do you suggest?
4. Which instructions require less information?
5. Are any tests not useful or difficulty to do? (Specify)
6. Should any other tests be added in?
7. How long does it take you to do the screen?
8. Other comments?

Comments collected from feedback sheets and reports were collated descriptively.

Patient and Public Involvement

Patients were involved in the design and monitoring of this study. Patients from the VISable stroke and vision panel were consulted when devising the study plan and conduct. Reports during the conduct of this study were circulated to the VISable panel for patient monitoring purposes.

Results

Completion rate

Two hundred and twenty-one stroke patients received both a VISA screening assessment and a reference vision assessment (during the period of September 2016 to February 2019). All elements of the VISA screen were attempted by 201 patients. VISA print was used with 121 patients from which complete data was available for 101 for analysis. The mean age of patients on stroke admission was 70.6 (SD 13.5), 46 were female and 54 male. The reported mean time of test duration was 23.5 minutes (SD 10.0).

For peer review only - http://bmjopen.bmj.com/site/about/guidelines.xhtml

VISA app was completed with 100 patients was a mean age of 63.4 (SD 13.4), of which 72 were male and 28 were female.

VISA print was fully completed by 91 patients, with the remaining 10 missing one or more elements (near vision n=5, distance vision n=5, ocular motility n=1, visual fields n=1, visual inattention n=9). The orthoptic vision assessment was fully completed by 90 patients, with the remaining 11 missing one or more elements (near vision n=8, visual inattention n=9). Reasons for inability to complete one or more of the elements were typically recorded as either cognitive impairment or fatigue. VISA app and orthoptic vision assessment were fully completed by all 100 patients. Missing data did not automatically result in failure for that section, thereby requiring referral. The reason for failure was taken into account; for example if a section was not completed due to fatigue this would not pragmatically have resulted in a referral but instead, a retest.

Referral agreement for VISA print

The agreement of whether to make a referral to specialist eye services based on the results of the VISA print versus those from orthoptic vision assessment had a Kappa value of 0.648 (substantial agreement) (95% CI 0.424 – 0.872).

Sensitivity of 97.67% and specificity of 60.00% were found. The positive and negative predictive values were 93.33% and 81.82% respectively. These calculations are outlined in Table 1. Agreement was found for 93 participants (nine had no visual impairment, 84 required referral because of failed screening) as outlined in Figure 1.

Table 1: Calculations of sensitivity, specificity and predictive values for VISA print

Positive i.e. pathologic n=86	
True positive, i.e. visual impairment present and referred	84
False negative, i.e. visual impairment present but not referred	2
Negative *i.e. normal n=15*	
False positive, i.e. visual impairment not present but referred	6
True negative, i.e. visual impairment not present and not	9
Output	

For peer review only - http://bmjopen.bmj.com/site/about/guidelines.xhtml

For peer review only

Sensitivity (true positive/true positive + false negative)	97.67% (95% CI: 91.85 – 99.72%)
Specificity (true negative/false positive + true negative)	60.00% (95% CI: 32.29 – 83.66%)
Positive predictive value (true positive/false positive + true positive)	93.33% (95% CI: 88.27 – 96.30%)
Negative predictive value (true negative/false negative + true negative)	81.82% (95% CI: 51.83 – 94.95%)

VISA print produced two false negative and six false positive results. Of the false negative results, both had ocular motility problems, of which one also had reduced near vision. The two ocular motility problems missed were asymptomatic minimal rotary nystagmus and limited elevation. The latter also had reduced near vision at 0.450 LogMAR. For false positive results, three with reduced near vison, two with ocular motility problems and one with both reduced near vision and visual inattention, were detected by screening and found not to be present by the orthoptic vision assessment. The referral relating to reduced near vision all detected N8 level of vision in one or both eyes. The referral relating to visual inattention were detected on the clock drawing element; it was noted by the examiner that the inaccurate completion was likely due to cognitive impairment. The ocular motility problems detected were reported as limitation of vertical gaze and nystagmus.

Test component agreement for VISA print

The agreements for the individual components between VISA print and orthoptic vision assessments are outlined in Table 2. The highest levels of agreement were produced for distance visual acuity (0.565) and visual fields (0.504), both with moderate agreement. The lowest level of agreement was produced for near visual acuity (0.236) and ocular motility (0.367), both with fair agreement. Low agreement for ocular motility related to high false positives and false negatives. In ten cases (one with multiple conditions) were not detected (false negative). These comprised of four defects of vertical movement (including one upgaze palsy, two restrictions of elevation and one V-pattern), three cases of nystagmus (including one minimal rotary nystagmus, one gaze-evoked and one end-point nystagmus), and four cases of reduced convergence. The low agreement with near visual acuity related to high false negatives where 23 cases were not detected – these comprised of ten with 0.4 LogMAR or better, nine between 0.4 and 0.5 LogMAR and three 0.6 LogMAR or worse.

For peer review only - http://bmjopen.bmj.com/site/about/guidelines.xhtml

Table 2: Summary of agreement between VISA print and orthoptic vision assessment for referral to specialist eye services and individual components

**Element of testing**	**Agreement**	**False negative**	**False positive**	**Kappa value (95% CI)**
Referral	93	2	6	0.648 (0.424 – 0.872)
Near visual acuity	65	23	12	0.236 (0.045 – 0.427)
Distance visual acuity	79	9	13	0.565 (0.405 – 0.725)
Ocular alignment	89	5	7	0.388 (0.110 – 0.667)
Ocular motility	72	10	19	0.367 (0.181 – 0.553)
Visual fields	76	7	18	0.504 (0.339 – 0.668)
Visual inattention	74	4	21	0.500 (0.340 – 0.659)

Referral agreement for VISA app

The agreement of whether to make a referral to specialist eye services based on the results of VISA app versus those from orthoptic vision assessment had a Kappa value of 0.690 (substantial agreement) (95% CI 0.528-0.851).

Sensitivity of 88.31% and specificity of 86.96% were calculated. The positive and negative predictive values were 95.77% and 68.97% respectively. These calculations are outlined in Table 3.

Table 3: Calculations of sensitivity, specificity and predictive values for VISA app

Positive i.e. pathologic n=77	
True positive, i.e. visual impairment present and referred	68
False negative, i.e. visual impairment present but not referred	9
Negative i.e. normal n=23	
False positive, i.e. visual impairment not present but referred	3
True negative, i.e. visual impairment not present and not	20
Output	
Sensitivity (true positive/true positive + false negative)	88.31%<br>(95% CI: 78.97 – 94.51%)

For peer review only - http://bmjopen.bmj.com/site/about/guidelines.xhtml

Specificity (true negative/false positive + true negative)	86.96%<br>(95%CI: 66.41 – 97.22%)
Positive predictive value (true positive/false positive + true positive)	95.77%<br>(95% CI: 88.72 – 98.49%)
Negative predictive value (true negative/false negative + true negative)	68.97%<br>(95% CI: 54.10 – 80.73%)

Agreement was found for 88 participants (20 had no visual impairment, 68 required referral because of failed screening) as outlined in Figure 1. VISA app produced nine false negative and three false positive results. Of the false negative results, four had slightly reduced near vision between 0.3 and 0.4 LogMAR, two had reduced distance vision of 0.3 LogMAR, two had mild visual inattention (one detected on clock cancellation and was the examiners judgement) and one had reduced near vision of 0.4 LogMAR and a visual field defect (partial right superior quadrantanopia detect on confrontation only, but not detected by formal perimetry using binocular Esterman). False positive results (one with reduced distance vision, one with a visual field defect and one with both a visual field defect and visual inattention) were detected by screening and found not to be present by the orthoptic vision assessment. The referral relating to reduce distance vision was 0.4 LogMAR. The referral relating to a visual field defect of general constriction and the visual inattention was detected on the longest line in the line bisection element. The other visual field defect detected was general constriction.

Test component agreement for VISA app

The agreements for the individual components between VISA app and orthoptic vision assessments are outlined in Table 4. The highest levels of agreement were produced for distance visual acuity (0.783) and visual fields (0.701), both with substantial agreement. The lowest level of agreement was produced for visual inattention (0.323) with fair agreement. The low agreement with visual inattention related to 16 false positives, of which 13 were detected with one of the three tests, 12 with line-bisection, 1 with clock drawing.

For peer review only - http://bmjopen.bmj.com/site/about/guidelines.xhtml

Table 4: Summary of agreement between VISA app and orthoptic vision assessment for referral to specialist eye services and individual components.

Element of testing	Agreement	False negative	False positive	Kappa value (95% CI)
Referral	88	9	3	0.690 (0.528 – 0.851)
Near visual acuity	77	19	3	0.416 (0.227 – 0.605)
Distance visual acuity	90	6	4	0.783 (0.656 – 0.910)
Visual fields	85	3	12	0.701 (0.564 – 0.838)
Visual inattention	78	6	16	0.323 (0.108 – 0.538)

Perimetry agreement

Twenty-five participants had formal perimetry using the binocular Esterman programme rather than confrontation. There was perfect agreement (1.0) of whether a visual field defect was present between the kinetic visual field test on VISA app versus formal perimetry using the binocular Esterman programme. Twenty-one had a visual field defect and four were found to have a normal visual field.

Process evaluation

Information from feedback sheets and detailed notes from interviews were compiled and grouped for type of feedback. Minimal feedback was obtained during the validation study. Feedback related to the duration of screening, presentation of tests on the app and referral guides. One stroke unit noted that VISA could take too long in the hyper-acute stage with unwell patients. Feedback on app presentation included a change to the clock drawing circle (to remove lines that might indicate time markers), change to the fixation target for the visual field test, additional of a nystagmus check on eye movement testing (in addition to its presence in the case history checklist) and ability to delete erroneous marks on the line bisection test. For referral guidance, feedback requested the addition of a refer / retest icon on the patient results page. Further feedback reported greater ease of screening with the app for those having to use their non-dominant hand because of upper limb motor impairment. Stroke survivors found it easier to respond using the touch screen than traditional pen and paper tasks when using their non-dominant hand.

For peer review only - http://bmjopen.bmj.com/site/about/guidelines.xhtml

Discussion

In this study, we present the VISA screening tool, performed by non-eye trained specialists, with validation results for the printed version and for the software app. Overall, referral had sensitivity and specificity of >88% and >60% respectively, positive and negative predictive values of >93% and >68% respectively, with substantial agreement between VISA screening and comprehensive orthoptic assessment of about Kappa 0.7. Agreement was lowest for eye movement screening, near visual acuity and visual inattention whereas all other individual sections showed higher levels of agreement. Process evaluation aided further refinement of VISA and, in particular, changes to presentation features on the app version.

When designing and using screening tools there is a balance between sensitivity and specificity for reliable detection of deficits. Low agreement in the VISA sections related to high false positive referrals where VISA screen indicated a fail for ocular motility or visual inattention. The orthoptic vision assessment confirmed ocular motility changes which were classed as 'normal' physiological eye movement patterns such age-related reduced elevation, and which alone would not have required referral. False positive referrals for visual inattention occurred where the patient failed to complete the section because of fatigue or cognitive impairment. Reduced visual acuity was always at a borderline level just above the fail threshold.

False negative referrals are important to consider; failed detection of significant deficits is to be avoided. Our results showed low numbers of false negatives which included failed detection of ocular motility defects, reduced visual acuity, visual inattention and visual field defect. The ocular motility defects were related to asymptomatic limited elevation and minimal nystagmus which would not have constituted referrals by orthoptic vision assessment. Reduced visual acuity, similar to the false positive results, was always close to the pass/fail threshold. Arguably, this is an ideal call for retest rather than refer. One case of mild visual inattention was not passed by VISA app where the diagnosis had been made by clinical observation. One visual field defect related to a peripheral field loss; a defect that could not be detected by the central testing area of VISA app.

Specificity was higher when using VISA app compared to VISA print. Mean age for the VISA app group was lower than the VISA print group with more male than female participants in the VISA app group. It is unlikely that age/sex differences affected agreement between the VISA print/app formats versus orthoptic assessment as all participants, by default of

For peer review only - http://bmjopen.bmj.com/site/about/guidelines.xhtml

meeting the inclusion criteria, were able to undergo both assessments. Differences are more likely due to the staff mix using VISA. VISA print was used solely by members of the stroke team and often without any formal vision training. VISA app was used by a mix of stroke team members but also orthoptists. Accuracy was likely enhanced by involvement of the latter. Referral agreements overall for decision on making a referral were 0.648 (VISA print) and 0.690 (VISA app), both indicating substantial agreement. It should be noted that Kappa is dependent on the base rate of the outcome being assessed, with calculated values being lower when the prevalence of the outcome is either very high or very low. Bruckner and Yoder suggest estimating overall accuracy using a combination of Kappa and base rate of outcome [20]. This method does not change the conclusions drawn here, as these outcomes with substantial agreement have estimated accuracy of at least 90% when base rate is taken into account, and those with moderate agreement at least 85% estimated accuracy.

VISA print and app provide a vision screen across the main categories of potential visual impairment following stroke. Besides a case history section, screening includes visual acuity, eye position and movements, visual fields and visual inattention. There are potential advantages for using either the manual tool or the app. Some clinicians and stroke survivors may prefer and respond better to use of traditional testing options inclusive of pen and paper tasks. The recording charts are completed during the testing period and can be entered in hospital case notes immediately. The app produces a PDF file of results which has to be printed before entry in hospital case notes. Conversely, the PDF file is an advantage for electronic hospital records. Further, the app provides a constant background illumination for screening assessments whereas the manual is used under variable lighting conditions dependent on wherever the screening is undertaken. The app uses a kinetic central visual field assessment that is run as a standardised test which reduces examiner bias – a bias that persists for confrontation visual field assessment.

When developing and validating a screening tool it is important that it is compared to a gold standard. In our UK services the gold standard is an orthoptic assessment undertaken on the acute stroke unit. The development and pilot of VISA followed a robust process [14]. In this follow-on validation study we considered the results of VISA versus the gold standard orthoptic assessment in evaluating construct and content validity. We further considered ecological validity through use of the tool by clinical (not research) stroke teams in the real-

For peer review only - http://bmjopen.bmj.com/site/about/guidelines.xhtml

world environment of busy acute stroke units in the UK National Health Service. Additionally, we sought specific feedback through process evaluation collecting feedback forms from stroke team clinicians and patient reports.

A systematic review of screening options for post-stroke visual impairment reported vision screening checklists and stroke screening tools (e.g. National Institute for Health Stroke Scale, FAST-AVVV, ABCD-E2) which include elements of vision assessment, however not all potential visual impairments are screened [21]. Past vision screening publications have reported the results of vision 'checklists' – lists of information gathered from questioning the patient, observations or from data documented in the case notes. The Vision In Stroke (VIS) reported checklist screening in 915 stroke survivors with sensitivity of 0.42, specificity of 0.52 and agreement against a reference standard of 0.428 (-0.048 to 0.019 CI: Kappa) [2, 12]. An Australian study reported the use of a checklist for detection of eye conditions and vision defects in 100 stroke survivors with 69% accuracy and intra-class correlation of 0.84 (0.77-0.89 CI) [22]. More recently, a vision screening app (available on android platforms) was developed for use with stroke survivors (StrokeVision app) with sections assessing visual acuity, visual fields and visual inattention [23]. This was validated with a cohort of 45 stroke survivors with sensitivities across the various sections of 50-79% and specificities of 87-98%. The specificities reported are higher than those from our VISA study but likely reflect the use of the StrokeVision app by fully trained research assistants versus the VISA completion by members of the stroke multidisciplinary team who only followed the in-built screening instructions.

Overall, vision tools/apps provide a more extensive screening of vision with greater accuracy than vision checklists. However, there are issues with how best to identify the presence of visual impairment through stroke team vision screening and specialist vision assessment [21, 24]. Even with screening measures in place there are also issues reported with provision of care and access to vision services for stroke survivors who have been identified as having vision problems [13].

An ideal stroke vision service follows recommendations from the National Clinical Guidelines for Stroke which specify orthoptists as core members of the acute stroke team and screen all stroke survivors prior to discharge [16]. Despite consistent findings that inclusion of vision services within the multi-disciplinary team (MDT) is highly beneficial, such visual assessment is not common and services are inconsistent throughout the UK. Stepped

For peer review only - http://bmjopen.bmj.com/site/about/guidelines.xhtml

models of care must be considered to meet the needs of stroke survivors against the context of local service capacity. Access to orthoptic services on acute stroke units enables faster provision of vision screening. The earlier assessment time-point reported for the IVIS study is important as it shows the feasibility and acceptability of early visual assessment within 3 days of stroke onset for at least half of stroke survivors and within 1 week of stroke onset for the majority [4]. Early detection of visual impairment is important. Although some cases of visual impairment will recover quickly, the majority do not. Moreover, there are few predictive factors for who will recover [4]. This prompt early detection, in turn, allows early detection of visual impairment and sharing of the functional significance of this with the patients, carers and stroke teams. Furthermore, early assessment leads to early intervention which has potential impact on general rehabilitation where visual function can be improved [2, 3, 10]. In the absence of orthoptic services, further stepped down models of care include the use of screening tools or screening checklists.

Such screening methods cannot replace the accuracy of a reference, specialist vision assessment. However, they serve an important purpose of obtaining a standardised screen in the absence of on-site specialist vision services and are better than no or non-standardised assessments by non-eye trained clinicians. In such instances, we advise the use of a screening tool. The advantages of VISA print and app are their validation in a real-world pragmatic study conducted in acute stroke units and used by non-eye trained clinicians. Clinicians used the in-built standalone instructions – designed to avoid the need of regular specialist vision training which can be difficult to access or provide. Further, the app is MHRA approved for clinical assessment; an important requirement for NHS adoption. The availability of VISA as a manual and as an app facilitates use alongside paper-based records or integration with electronic patient record systems.

Vision checklists have been shown to have a low sensitivity and specificity, and an over-reliance on the report of visual symptoms [2, 12]. The VISA print and app offer an intermediate measure between vision checklists and orthoptic specialist vision services with greater accuracy than vision checklists but lacking the accuracy of orthoptic assessments and the immediate access to management of visual problems provided by orthoptic stroke unit services. The VISA print/app does not preclude the use of vision checklists however, for some stroke survivors who are very unwell acutely and/or lack sufficient cognition and

For peer review only - http://bmjopen.bmj.com/site/about/guidelines.xhtml

communication, simpler vision checklists are quick and easy to use in such circumstances and remain more accurate than no vision screen at all.

There are some limitations to consider for this study. The VISA screening tool was used on acute stroke units. There was no validation of the tool in community settings. However, there is little reason to think it would be of less or use or accuracy when used in other stroke settings. Test-retest and inter-rater variability of the VISA screening tool were not evaluated during this study as this would have caused too high a burden of assessment on participants. Information on education level, stroke type, stroke severity and ocular history were not obtained for this study. These sources of information were not considered essential to this study as the primary aim was to determine if VISA could detect visual impairment regardless of patient/stroke demographics and regardless of whether visual impairment was new or pre-existent. These aspects would provide potentially useful discussion in a future implementation study of VISA. A further limitation is that we included a convenience sample of stroke survivors in this study. The study was designed as a pragmatic clinical study to fit in with daily clinical practice and with minimal disruption to service and care on the acute stroke unit. As a result the stroke team were potentially more likely to screen stroke survivors at risk for visual impairment. This may explain the higher prevalence rate of visual impairment for this study (85% VISA print and 77% VISA app) than that reported in a recent epidemiology study (73%) [4].

Conclusions

Validation of the VISA screening tool in either print or app format shows improved detection accuracy for detection of stroke-related visual impairment by clinicians involved in stroke care who are not specialists in vision problems and lack formal eye training. Where early visual impairment detection occurs, this facilitates prompt referral with fewer false positives and negatives. Clinicians reported acceptability of the VISA screening tool for is use in screening for presence of vision problems in stroke survivors. Referral sensitivity of >88% and specificity of >60% were found for the VISA screening with substantial inter-rater agreement for referral between VISA screening and specialist vision assessments. The VISA screening tool provides a standardised and validated method to screen for visual problems

For peer review only - http://bmjopen.bmj.com/site/about/guidelines.xhtml

following stroke and may further be of potential use for visual screening in other care settings such as neuro-rehabilitation.

Acknowledgements

We thank the VISable panel for their involvement in the design and monitoring of this study. We thank the patients and staff at Arrowe Park Hospital, Bradford Teaching Hospital, Manchester University Hospital, Salford Royal Hospital and the Walton Centre; specifically Felicity Hale, Jennifer Lawrence, Sam Campbell, Claire Fowler, Imtiaz Nazir, Laura Stubbs, Abigail Kerr, Stephanie Wolff, Kim Cochrane, Tamsin Steward, Ella Henry, Carmel Noonan and Hayley Draper.

References

1. Hepworth LR, Rowe FJ, Walker MF, et al. Post-stroke visual impairment: a systematic literature review of types and recovery of visual conditions. *Ophthalmology Research* 2016;5(1). doi:10.9734/OR/2016/21767
2. Rowe F, VIS writing group. Vision In Stroke cohort: profile overview of visual impairment. *Brain and Behavior* 2017. doi:10.1002/brb3.771
3. Jones SA, Shinton RA. Improving outcome in stroke patients with visual problems. *Age and Ageing* 2006;35(6):560-5
4. Rowe FJ, Hepworth LR, Howard C, et al. High incidence and prevalence of visual problems after acute stroke: an epidemiology study with implications for service delivery. *PLoS One* 2019;14(3). doi:10.1371/journal.pone.0213035
5. Stroke Association. State of the nation: stroke statistics 2018 [Available from: https://www.stroke.org.uk/system/files/sotn_2018.pdf.
6. Hepworth LR, Rowe FJ. Visual impairment following stroke - the impact on quality of life: a systematic review. *Ophthalmology Research* 2016;5(2):1-15
7. Tsai S-Y, Cheng C-Y, Hsu W-M, et al. Association between visual impairment and depression in the elderly. *Journal of Formosan Medical Association* 2003;102(2):86-90
8. Chen CS, Lee AW, Clarke G, et al. Vision-related quality of life in patients with complete homonymous hemianopia post stroke. *Topics in Stroke Rehabilitation* 2009;16(6):445-53
9. Gall C, Lucklum J, Sabel BA, et al. Vision- and health-related quality of life in patients with visual field loss after postchiasmatic lesions. *Investigative Ophthalmology and Vision Science* 2009;50(6):2765-76
10. Hanna KL, Hepworth LR, Rowe FJ. The treatment methods for post-stroke visual impairment: a systematic review. *Brain and Behavior* 2017;7(5). doi:10.1002/brb3.682
11. Rowe F, Walker M, Rockliffe J, et al. Delivery of high quality stroke and vision care: experiences of UK services. *Disability and Rehabilitation* 2016;38(8):813-7
12. Rowe FJ, VIS Group UK. Accuracy of referrals for visual assessment in a stroke population. *Eye* 2011;25(2):161-7
13. Rowe FJ, Walker M, Rockliffe J, et al. Care provision for poststroke visual impairment. *Journal of Stroke and Cerebrovascular Diseases* 2015;24(6):1131-44
14. Rowe FJ, Hepworth LR, Hanna KL, et al. Visual Impairment Screening Assessment (VISA) tool: pilot validation. *BMJ Open* 2018. doi:10.1136/bmjopen-2017-020562

For peer review only - http://bmjopen.bmj.com/site/about/guidelines.xhtml

15. Cohen JF, Korevaar DA, Altman DG, et al. STARD 2015 guidelines for reporting diagnostic accuracy studies: explanation and elaboration. *BMJ Open* 2016;6(e012799). doi:10.1136/bmjopen-2016-012799
16. Intercollegiate Stroke Working Party. National clinical guideline for stroke. 5th ed. London: Royal College of Physicians; 2016.
17. British and Irish Orthoptic Society. Compentency standards and professional practice guidelines: extended practioner role London: British and Irish Orthoptic Society,, 2018.
18. Holey EA, Feeley JL, Dixon J, et al. An exploration of the use of simple statistics to measure consensus and stability in Delphi studies. *BMC Medical Research Methodology* 2007;7(52). doi:10.1186/1471-2288-7-52
19. Bland JM, Altman DG. Statistical methods for assessing agreement between two methods of clinical measurement. *Lancet* 1986;327(8476):307-10
20. Bruckner CT, Yoder P. Interpreting Kappa in observational research: baserate matters. *American Journal of Mental Retardation* 2006;111(6):433-41
21. Hanna KL, Hepworth LR, Rowe FJ. Screening methods for post-stroke visual impairment: a systematic review. *Disability and Rehabilitation* 2017;39(25):2531-43
22. Jolly N, Macfarlane A, Heard R. Towards gaining the best information about vision to assist the recovery of a patient with stroke. *Strabismus* 2013;21(2):145-9
23. Quinn TJ, Livingstone I, Weir A, et al. Accuracy and feasibility of an android-based digital assessment tool for post stroke visual disorders - the StrokeVision app. *Front Neurology* 2018;9(146). doi:10.3389/fneur.2018.00146
24. Rowe FJ. Who sees visual impairment following stroke? *Strabismus* 2010;18(2):37-40

Figure 1: Flow diagram of participant outcome for VISA screening and orthoptic full assessment.

For peer review only

Figure 1: Flow diagram of participant outcome for VISA screening and orthoptic full assessment.

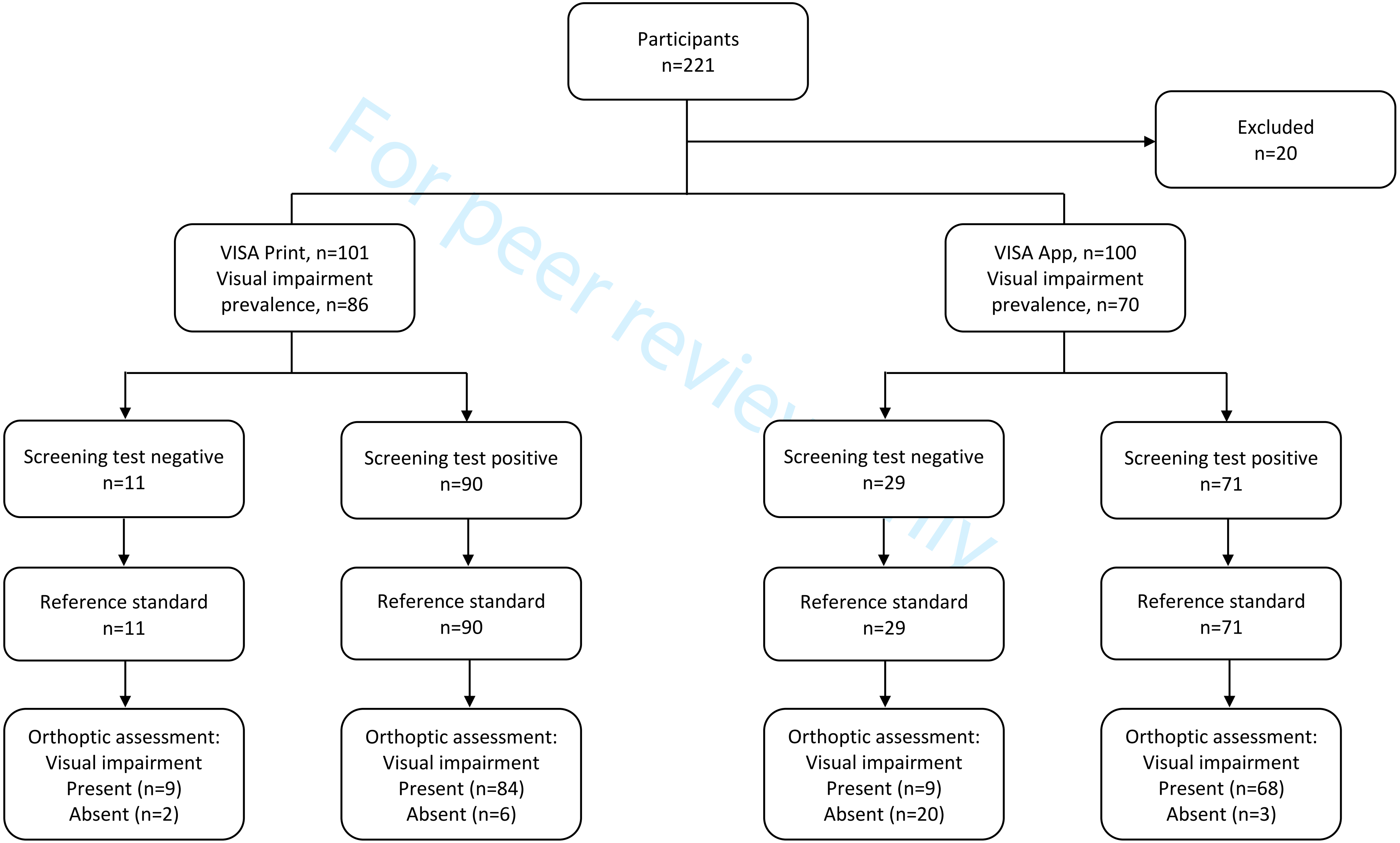

For peer review only - http://bmjopen.bmj.com/site/about/guidelines.xhtml

Section & Topic	No	Item	Reported on page #
**TITLE OR ABSTRACT**			
	**1**	Identification as a study of diagnostic accuracy using at least one measure of accuracy (such as sensitivity, specificity, predictive values, or AUC)	1
**ABSTRACT**			
	**2**	Structured summary of study design, methods, results, and conclusions (for specific guidance, see STARD for Abstracts)	2
**INTRODUCTION**			
	**3**	Scientific and clinical background, including the intended use and clinical role of the index test	3
	**4**	Study objectives and hypotheses	3
**METHODS**			
Study design	**5**	Whether data collection was planned before the index test and reference standard were performed (prospective study) or after (retrospective study)	3
Participants	**6**	Eligibility criteria	3-4
	**7**	On what basis potentially eligible participants were identified (such as symptoms, results from previous tests, inclusion in registry)	4
	**8**	Where and when potentially eligible participants were identified (setting, location and dates)	3-4
	**9**	Whether participants formed a consecutive, random or convenience series	4
Test methods	**10a**	Index test, in sufficient detail to allow replication	4
	**10b**	Reference standard, in sufficient detail to allow replication	4
	**11**	Rationale for choosing the reference standard (if alternatives exist)	N/A
	**12a**	Definition of and rationale for test positivity cut-offs or result categories of the index test, distinguishing pre-specified from exploratory	3 – in previously published paper
	**12b**	Definition of and rationale for test positivity cut-offs or result categories of the reference standard, distinguishing pre-specified from exploratory	5
	**13a**	Whether clinical information and reference standard results were available to the performers/readers of the index test	5
	**13b**	Whether clinical information and index test results were available to the assessors of the reference standard	5
Analysis	**14**	Methods for estimating or comparing measures of diagnostic accuracy	5
	**15**	How indeterminate index test or reference standard results were handled	6
	**16**	How missing data on the index test and reference standard were handled	6
	**17**	Any analyses of variability in diagnostic accuracy, distinguishing pre-specified from exploratory	N/A
	**18**	Intended sample size and how it was determined	5
**RESULTS**			
Participants	**19**	Flow of participants, using a diagram	Figure 1
	**20**	Baseline demographic and clinical characteristics of participants	6
	**21a**	Distribution of severity of disease in those with the target condition	7-8
	**21b**	Distribution of alternative diagnoses in those without the target condition	N/A
	**22**	Time interval and any clinical interventions between index test and reference standard	5
Test results	**23**	Cross tabulation of the index test results (or their distribution) by the results of the reference standard	Table 1 and 3
	**24**	Estimates of diagnostic accuracy and their precision (such as 95% confidence intervals)	6-7 and Tables 1 and 3
	**25**	Any adverse events from performing the index test or the reference standard	9
**DISCUSSION**			
	**26**	Study limitations, including sources of potential bias, statistical uncertainty, and generalisability	12
	**27**	Implications for practice, including the intended use and clinical role of the index test	11-12
**OTHER INFORMATION**			
	**28**	Registration number and name of registry	N/A
	**29**	Where the full study protocol can be accessed	N/A
	**30**	Sources of funding and other support; role of funders	1

For peer review only

For peer review only - http://bmjopen.bmj.com/site/about/guidelines.xhtml

For peer review only
